# Interplay of Oxidative Stress, Inflammation, and Autophagy: Their Role in Tissue Injury of the Heart, Liver, and Kidney

**DOI:** 10.1155/2018/2090813

**Published:** 2018-03-22

**Authors:** Partha Mukhopadhyay, Nabil Eid, Mohamed A. Abdelmegeed, Aditya Sen

**Affiliations:** ^1^Laboratory of Cardiovascular Physiology and Tissue Injury, NIAAA, National Institutes of Health, Bethesda, MD 20852, USA; ^2^Division of Life Sciences, Department of Anatomy and Cell Biology, Osaka Medical College, Takatsuki, Osaka 569-8686, Japan; ^3^Section of Molecular Pharmacology and Toxicology, Laboratory of Membrane Biochemistry and Biophysics, National Institute on Alcohol Abuse and Alcoholism, Bethesda, MD, USA; ^4^Department of Biochemistry and Molecular Biology, Uniformed Services University, Bethesda, MD 20814, USA

Oxidative/nitrosative stress, inflammation, and autophagy roles have been described in many studies as prominent factors in mediating many pathological alterations, in response to toxic agents or in disease states [1]; however, it is not well characterized whether there is an interplay between these three factors or any combination of them, in addition to their sole effect, in mediating the harmful mechanisms of pathological alterations particularly in the heart, liver, and kidney. These harmful mechanisms might be identified directly via exposure to various insults or indirectly via evaluating the mechanism(s) of the prevention of these pathological alterations or by both methods. It was our intention in this special issue to invite new insights and to shed a light on this very interesting topic that would encourage scientists to identify new therapeutic targets for various diseases, in the context of oxidative stress, inflammation, and autophagy, and to tackle them in hope of developing better treatments.

It is well established that there is a balance between reactive oxygen and nitrogen species (ROS and RNS, resp.) production and removal in the body under normal physiological conditions and that basal low levels of ROS/RNS is vital for cell signaling and cell survival [2]. However, upon exposure to chemicals or toxic agents such as alcohol, acetaminophen, and cisplatin, or under disease state such as diabetes, cancer, inflammatory diseases, and ischemia reperfusion, to name a few, ROS/RNS may be produced in excessive amounts, beyond the antioxidant cell defense capacity, from many sources within the cell such as cytochromes P450 2E1 and 4A, nicotinamide adenine dinucleotide phosphate-oxidase (NADPH oxidase), xanthine oxidase, and mitochondrial electron transport chain [3–5]. ROS/RNS can also be produced in response to inflammation and immune cell activation [6]. The increased levels of ROS/RNS may induce their deleterious effects in the cells by damaging and/or modifying proteins, DNA, lipids, and vitamins, contributing to disturbance of their normal functions, which might be essential for cell survival, leading to the development and/or progression of tissue injury.

Although oxidative stress might be induced by inflammation, inflammation is also one of the most common outcomes of oxidative stress. It has been suggested that increased oxidative stress levels might stimulate the expression of chemokines and cytokines leading to increased inflammation [7]. As with oxidative stress, inflammation is also part of the host defense mechanism and might be important for maintain vital functions; however, excessive inflammation might cause cellular damage and/or death.

Autophagy is an essential process for normal cell homeostasis. Many forms of autophagy have been identified such as macroautophagy, microautophagy, and chaperone-mediated autophagy. Autophagy is being considered as one of the cellular defense against increased oxidative stress as well as other diverse cell stress conditions [8]. Intriguingly, autophagy has been reported to regulate oxidative stress and inflammation signaling and formation [9].

Thus, it seems that there might be an interplay between oxidative stress, inflammation, and autophagy and that unchecked oxidative stress, unresolved inflammation, and disturbed autophagy are common features in many diseases, upon exposure to toxic chemicals, and even exposure to unhealthy dietary habits. The use of antioxidant and anti-inflammatory whether naturals or chemicals, particularly naturals, might be an easy and effective method to prevent disease development and/or progression, as these agents might consequently prevent disruption of autophagy process.

The articles included in this special issue review addressed many of the goals of this issue as they provide better understanding in some of the underlying mechanisms of some of the important pathological changes that occur during tissue injury upon exposure to injurious insults as well as providing some potential preventive and/or therapeutic approaches to prevent tissue injury, particularly by natural compounds. There are two articles involving the beneficial effects of baicalin, which is a main bioactive component of *Scutellaria baicalensis* Georgi (*S. baicalensis*), used as a traditional Chinese herbal medicine, and has been recently a focus of many studies due to its seemingly beneficial effects. The first article, by using in vivo and in vitro models, suggested that baicalin ameliorates biliary duct ligation-induced experimental liver fibrosis alleviating inflammation, oxidative stress, and apoptosis. Another study reported that baicalin attenuated subarachnoid hemorrhage in mice via decreasing inflammation (inhibited microglial activation), oxidative damage, and brain edema. Apigenin, an abundant dietary flavonoid that can be found in many fruits and vegetables, was reported by one of the articles to alleviate myocardial toxicity in a mouse model induced by endotoxin via the modulation of oxidative stress, inflammation, and autophagy. Daidzein, another naturally occurring compound belong to isoflavones that can be found in soybeans and other legumes, by using both in vivo and in vitro models, was shown to improve kidney regeneration in a cisplatin-induced nephrotoxicity model through decreased levels of nitroxidative stress, inflammation, and apoptosis. In another article related to heart diseases, ischemia preconditioning was suggested to alleviate cardiac infarction via the increased expression of immediate early response gene (IEX-1), which might decrease cardiac apoptosis and necrosis via decreased intracellular ROS accumulation. Rhodiola sacra, a genotype of Rhodiola species, a famous genus of Chinese medicinal herb, when combined with exercise was shown by one of the articles of this issue to enhance mitochondrial quality control leading to improvement of exercise capacity and decrease exhaustive exercise-induced skeletal and cardiac muscle damage. Another article suggested a potentially protective role of granulocyte colony-stimulating factor (G-CSF) against neonatal brain suffering from bacteria-induced meningitis, possibly via a selective therapeutic action site of G-CSF through epigenetic histone modification particularly in the *TNFA* gene promotor. There was also an article suggesting a protective role of exogenous hydrogen sulfide against the development of acute kidney injury in response to LPS via the inhibition of inflammation and oxidative stress in a mouse model and blood samples from patients. An interesting study reported that bone marrow-derived mesenchymal stem cells (BMSCs) may have a therapeutic potential against sepsis by increasing Parkin-related mitophagy and decreasing mitochondrial oxidative stress leading to restriction of inflammasome activation in macrophages in a cecal ligation and puncture (CLP) mouse model. This might be a crucial mechanism for MSCs to combat sepsis in various models. Finally, the last article presented the development of cardiac autonomic neuropathy (CAN) and the early signaling changes in the myocardium as early consequences of mild metabolic challenge without significant changes on gross cardiac structure/function and the absence of signs of diabetes or impaired glucose tolerance. They also highlighted a potential corrective role for metformin and pioglitazone, which are not related to their blood glucose lowering effect and evaluated the effect of dietary interventions after a period of high caloric diet.

Taken together, we believe that these contributions advance the current knowledge about the pathophysiology of tissue injury with emphasis on the role of oxidative stress, inflammation, and autophagy and thus might help developing novel pharmacotherapeutic strategies for disease control and management.

Finally, we would like to thank all the contributors to this special issue for their participation and interest and many others who submitted but we could not accommodate.

*Partha Mukhopadhyay* *Nabil Eid* *Mohamed A. Abdelmegeed* *Aditya Sen*
